# Supplementary material for: Empirical vs. light-use efficiency modelling for estimating carbon fluxes in a mid-succession ecosystem developed on abandoned karst grassland
Source: PLoS One. 2020 Aug 7;15(8):e0237351. doi: 10.1371/journal.pone.0237351 (PMC7413509; doi:10.1371/journal.pone.0237351)
Supplement: Attachment — Submitted filename: Response to Reviewers.docx [file pone.0237351.s001.docx]

We would like to thank the editor and the two reviewers for their valuable comments, that certainly improved the quality of our paper. Please find below a point by point response to all issues raised during the review process. We successfully addressed each of the comments in the manuscript, and changes can be easily noticed in the revised manuscript labelled “Revised Manuscript with Track Changes”.

**Editor’s comments regarding the journal’s requirements**

1. Please ensure that your manuscript meets PLOS ONE's style requirements, including those for file naming.

*Author response:* We have carefully checked the requirements of PLOS ONE, and made the changes to meet these requirements. For instance, we have now removed the zip code and added the department name on the title page, changed the naming of figure panels in the figure legend, table legends have been moved below tables, and all borders were added to tables.

Regarding file naming, we have now checked, and in our revised submission, we named all files according to the requirements. Figure names were also checked and meet the requirements of the journal. Figures resubmitted were also checked using the PACE engine.

Heading numbers were also removed as seen in the journal’s main body format requirement.

2. We note that you have stated that you will provide repository information for your data at acceptance. Should your manuscript be accepted for publication, we will hold it until you provide the relevant accession numbers or DOIs necessary to access your data. If you wish to make changes to your Data Availability statement, please describe these changes in your cover letter and we will update your Data Availability statement to reflect the information you provide.

*Author response*: Thank you for this reminder. The data used in the paper has now been published on Figshare, and is publicly available at the following DOI: <https://doi.org/10.6084/m9.figshare.12520778.v1> .

3. We note that Figure 1 in your submission contains map and satellite images which may be copyrighted. All PLOS content is published under the Creative Commons Attribution License (CC BY 4.0), which means that the manuscript, images, and Supporting Information files will be freely available online, and any third party is permitted to access, download, copy, distribute, and use these materials in any way, even commercially, with proper attribution.

*Author response:* We are very sorry that we did not write the source of the basemaps used, and we thank you very much for pointing that out. We have now put the correct reference of the basemaps used in the updated Figure 1.

Originally, one of the base maps was from National geographic, and we replaced it now with Open street maps (<https://www.openstreetmap.org/copyright>). The other basemap is a digital orthophoto produced by the Surveying and Mapping Authority of the Republic of Slovenia (GURS) (<https://www.e-prostor.gov.si/access-to-geodetic-data/ordering-data/#tab2-1791>). Both basemaps are distributed under creative commons with unrestricted re-use, either non-commercially or commercially, as you can see in the links between brackets.

Therefore, I have now added the licensing type on the revised Figure 1.

**Comments of the Reviewer 1**

This study estimated GPP and NEE based on VI empirical models and compared the estimation accuracy of GPP between empirical models and VPM model in a mid-succession ecosystem developed on abandoned karst grassland. Similar attempts have been increasingly made in recent years. An interesting finding is that enhanced vegetation index was the best proxy for NEE. This study is well designed, and the details of this study are well described. However, I still have several comments that need to be clarified.

1. It’s not clear to me why the authors used “in-sample” and “out-of-sample”? They are the training dataset and validation dataset, respectively?

*Author response:* We thank the reviewer for pointing out this inconsistency in our writing. We actually meant the same thing by “in-sample” and “training dataset” for instance. We have now corrected this throughout the manuscript, for consistency, and used only “train” and “test” instead of “in-sample” and “out-of-sample”.

2. Why did not you use independent data to validate the accuracy of VPM model? Although VPM GPP can be estimated directly by using several parameters, I think the validation is necessary.

*Author response:* We thank the reviewer for this valuable comment. If we did not have to apply correction factors, there would be no need to split the dataset because all the observed values would have been independent dataset used to validate the model through the calculation of accuracy metrics. However, the fact that we needed to correct the estimated values with a correction factor determined using regression makes the reviewer’s suggestion very useful.

Therefore, we have now used the multiple data splitting for finalizing the VPM model as used in our study (obtaining the correction factor using the training the dataset), and validating the model with the test dataset by the same occasion. The reason why we used the multiple data splitting is to overcome the limitation of the small dataset size.

3. Line 257, the subsection “3.3. Best models selection” only has one sentence, this absolutely needs to be improved and more descriptions are needed.

*Author response:* This is a very useful comment, and we are sorry for having overlooked this section. We have now added some more description, regarding why the best models were said to be best, and we also compared the best empirical model with the VPM model.

Thanks for highlighting this problem.

4. In discussion section, adding subsection will improve the readability.

*Author response:* We thank the reviewer for this comment which will certainly improve the readability of our paper. We have now split the discussion into 5 sections accordingly, which are: **4.1.** Empirical relationship between carbon fluxes and VI ; **4.2.** fAPAR, LUEmax and modifiers in the VPM model ; **4.3.** Other challenges of the VPM model ; **4.4.** VPM vs Empirical model ; **4.5.** Limitations of the study.

5. Many studies have demonstrated the feasibility of using VI, especially the EVI, and VPM model to estimate GPP. What are the differences in your research? What are the limitations of your research methods? Why EVI can be used to estimate NEE directly in your study? I think the above issues need to be further analyzed in the discussion.

*Author response:* We have now addressed these different issues in the discussion. Additionally, some of these issues which were already addressed before are more visible now after splitting the discussion into sections. The changes can be seen throughout the discussion section. We have now added a separate section that addresses the limitations of the study (section 4.5).

We thank the reviewer for this important comment.

6. Lines, 280, 289, 299, 300, 305, please check whether the citation format of the reference is correct.

*Author response:* Thank you very much for pointing out this issue that was also raised by the Reviewer 2. We have now reviewed the citation style so that it is scientifically correct. Wherever possible, we changed the sentence into passive voice and added the reference at the end, and when this was not possible, we added the author’s name before the shortened citation at the beginning of the sentence.

**Comments of the Reviewer 2**

The study compared the model performance of simple remote-based gross primary production (GPP) models and vegetation production model (VPM) with tower-based GPP estimation from EC observations in an abandoned karst grassland. However, some flaws need further improvement. At least, current manuscript is rough both in innovation and expression of results.

**Major points:**

1. Abstract: The first two sentences are off the point you want to study, because this study is a case study. Thus, maybe a bit better start is a simple and clear introduction for the importance and particularity of your research site.

*Author response:* This is a very good suggestion, and we thank the reviewer for making it. We now replaced the two sentences, talking instead about the importance of karst systems with regards to their carbon budget, and the fact that they can also emit an important amount of CO_2_ depending on whether or not there is a vegetation and the type of vegetation, hence underlining the importance of understanding the contribution of ecosystems to the carbon budget of karst areas.
We further added a related sentence in the introduction, so that everything written in the abstract has a source in the paper itself.

2. Introduction (Lines 47-55): the same problem with the point 1, process-based models and machine learning models are outside of your research. Thus, focus the introduction on the VI-based models and LUE models for GPP estimations.

*Author response:* Yes. We agree with this suggestion, that removes unnecessary developments, and keeps the introduction straight to the point. Accordingly, we removed the process-based models machine learning models from the introduction, and the introduction is more focused now on what was addressed in our study.

3. Results (Lines 257-259): Obviously, results of the best models selection are not showed in this part. Add some useful information in this part. In addition, considering a better model selection method because a simple comparison of statistical index (R2 and RMSE) is easy to make errors. For example, actually, it's hard to know which model is better for two models with similar R2, just like 0.82 and 0.8, or 0.73 and 0.72. (Fig. 3).

*Author response:* We thank the reviewer for this valuable comment. It is true that EVI and SAVI performed very similarly. We now computed the corrected AIC (or the AICc) which is ideal for model comparison for low sample sizes, while converging towards AIC for large samples. Therefore, the choice of AICc is the fact that it is suitable both for small and large sample sizes. AICc differences (ΔAICc) were computed between each candidate model and the model with the lowest AICc to support the other accuracy metrics in choosing the best model. According to Burnham and Anderson (2002) [reference included in the revised manuscript], a model can be said to be better than the other when the AICc difference is higher than 2. In this regard, while highlighting EVI as the best proxy for GPP for having the highest r² and the lowest rmse and AICc, we also highlighted the fact that based on ΔAICc < 2, SAVI performed similarly as EVI. For NEE however, the difference between EVI and SAVI was significant. More can be read about this in the revised manuscript.

**Minor points:**

1. Methods: add some information for your decision of footprint for EC-observations.

*Author response:* At first, we did not include any information about footprint analyses in our paper because the coauthor performed the analyses in a previous study that we referred to in our paper. However, we believe your suggestion will make the section of the study area easily understandable without necessarily going to the other paper. Therefore, we have now added a concise information about the footprint, especially the method used and the estimated average distance from where 90% of the fluxes were monitored by the tower.

2. Lines 95-97: “could be found in [24]”, it is a wrong way for references marking. You can rewrite it as “could be found in a previous study [24]”. So many similar references errors throughout the text, please check and revise.

*Author response:* Thank you for pointing out these errors in our referencing style. We corrected all the citations accordingly.
