# Supplementary material for: Empirical vs. light-use efficiency modelling for estimating carbon fluxes in a mid-succession ecosystem developed on abandoned karst grassland
Source: PLoS One. 2020 Aug 7;15(8):e0237351. doi: 10.1371/journal.pone.0237351 (PMC7413509; doi:10.1371/journal.pone.0237351)
Supplement: Attachment — Submitted filename: Response to Reviewers.docx [file pone.0237351.s002.docx]

We would like to thank the reviewers for their valuable comments, that has leveled our paper up in terms of consistency and coherence. Please find below a point by point response to all issues raised during the second round of review. We successfully addressed each of the comments in the manuscript, and changes can be noticed easily in the revised manuscript labelled “Revised Manuscript with Track Changes”.

**Comments of the Reviewer 1**

The author has solved almost all the problems I have raised. The manuscript in the present form has been greatly improved. There are two minor problems:

1. Lines 20-21: This sentence ‘The CO2 released can be absorbed again for photosynthesis if there is a good vegetation cover on the site’ seems unnecessary.

*Author response:* We thank the reviewer and have removed this unnecessary sentence that makes the abstract more concise.

2. Lines 292-296: These two paragraphs still have only one sentence, and they can be combined with the first paragraph (Lines 283-291).

*Author response:* This is absolutely a good suggestion, and we have joined these two paragraphs to the bigger previous one instead of having one sentence paragraphs which is not a good practice. We are very grateful to the reviewer for these valuable comments and suggestions

**Comments of the Reviewer 2**

The authors have addressed some of my concerns. However, some flaws need to be improved.

**Major points:**

1. Some paragraphs are not well organized. Some examples:

(1) in the section of “Study site” (Lines 71-92), I think, there is no need to divide it into three segments.

(2) Line 112: it belongs to your results from data acquisition, thus should not appear in the methods section.

(3) Data analysis (Lines 187-210): can be expressed in only one paragraph, no need for three.

(4) Best models selection (Lines 283-286): it belongs to “methods”, not “Results”.

(5) Limitations of the study (Lines 407-420): express it in only one paragraph.

………………

Please check other similar questions.

*Author response:* We thank the reviewer for these very important suggestions. We have now put together any unnecessarily split paragraphs (1), (3), (5) and other similar issues that we noticed by reading again the entire manuscript. The reviewer 1 also made such suggestions that we have taken into account. We have moved the text on model selection from results to integrate it in methods where it belongs (4). We have also moved the Figure 2 of Eddy covariance and vegetation indices to the results section and added some description to the newly created section. Additionally, we had the paper read by one peer, and considered his useful suggestions on language and phrasings as well.

With these changes, the manuscript is now more consistent and coherent, and we thank the reviewer for his useful comments.

2. Empirical models: in this study, why a simple quadratic model was selected to estimate GPP and NEE in this site, not others, just like simple linear regression, or exponential regression and so on? After multiple model comparisons? Or based on previous studies? Please specify it in the text.

*Author response:* It is true that we forgot to mention the reason for choosing a quadratic model. In fact, it was based on multiple model comparisons guided by the relationship observed between GPP or NEE and vegetation indices. We have now added this information in the methods sections where we presented the empirical model.

**Minor points:**

1. References: so many errors in references marking, e.g., line 88, line 94, line 105, line 284…., Be careful, and not to make such a low-grade mistake again.

*Author response:* We thank the reviewer for pointing out these citation issues. We thought it was okay to write the author’s name when we are referring to their model for instance. However, on the suggestion of the reviewer, we have now removed all author names, and rephrased differently either by using the phrasing “as in a previous study”, or by using passive voice and adding the reference at the end of the sentence. We have checked all citations in the text, and they are now all styled consistently, and comply with the citation style of the PLOS One journal.

2. Title of the section (or sub-section): Don't be too simple to accurately express your idea. e.g., Models (Line 130, GPP or NEE estimation models?), Empirical model (Line 212, Empirical model performance (in GPP or NEE estimation)?), fAPAR (Line 231, fAPAR estimation?).

*Author response:* We acknowledge that some of our titles are too simple and not clear enough. In this regard, we included the suggestions of the reviewer and have checked all titles across the document to make them more explicit. Apart from what was explicitly suggested by the reviewer, we also changed for instance the title “VPM model” to “VPM model performance in estimating GPP” (Line 256 of manuscript with track changes); the title “LUE_max_” to “Estimated LUE_max_” (Line 284 of manuscript with track changes).
